# Impact of temporal correlations on high risk outbreaks of independent and cooperative SIR dynamics

**DOI:** 10.1371/journal.pone.0253563

**Published:** 2021-07-20

**Authors:** Sina Sajjadi, Mohammad Reza Ejtehadi, Fakhteh Ghanbarnejad

**Affiliations:** Department of Physics, Sharif University of Technology, Tehran, Iran; Universidad Rey Juan Carlos, SPAIN

## Abstract

We first propose a quantitative approach to detect high risk outbreaks of independent and coinfective SIR dynamics on three empirical networks: a school, a conference and a hospital contact network. This measurement is based on the k-means clustering method and identifies *proper samples* for calculating the *mean outbreak size* and *the outbreak probability*. Then we systematically study the impact of different temporal correlations on high risk outbreaks over the original and differently shuffled counterparts of each network. We observe that, on the one hand, in the coinfection process, randomization of the sequence of the events increases the mean outbreak size of high-risk cases. On the other hand, these correlations do not have a consistent effect on the independent infection dynamics, and can either decrease or increase this mean. Randomization of the daily pattern correlations has no strong impact on the size of the outbreak in either the coinfection or the independent spreading cases. We also observe that an increase in the mean outbreak size does not always coincide with an increase in the outbreak probability; therefore, we argue that merely considering the mean outbreak size of *all realizations* may lead us into falsely estimating the outbreak risks. Our results suggest that some sort of contact randomization in the organizational level in schools, events or hospitals might help to suppress the spreading dynamics while the risk of an outbreak is high.

## Introduction

Infectious diseases have had drastic impacts on human health throughout history, resulting in major social and economic disruptions [1]. Through mathematical models, a better understanding and predictions on such phenomena has been achieved [2, 3]. The Susceptible-Infectious-Recovered (SIR) model [4] is one of the most basic and common models for describing and predicting the epidemics of contagious diseases. This model and its variations have been developed to model patterns of spreading dynamics in different scenarios. For instance, some models discuss how contact networks of the host population can alter the spreading, e.g., its epidemic threshold [5–12]. Some other works improved models by considering temporality of the contacts [13–15] and some studies focus on impact of temporal correlations on spreading dynamics [16, 17]. Moreover, some models consider the case of coinfective diseases, i.e., the infection of one disease altering the chance of infection by another. Such studies handled cooperative or competitive spreading dynamics in the mean field approximation and on complex networks with different topologies [18–23].

Despite these achievements, we lack a quantitative method to systematically measure the effect of the network temporal correlations on the spreading dynamics; especially when two or more spreading processes interact.

The increasing amount of empirical contact data, better computational performance, and our interest in understanding real-world situations, motivated us to propose a quantitative measurement for studying the impact of temporal correlations of the empirical networks on independent and cooperative SIR dynamics. To this end, we ran the independent and coupled SIR model on three empirical temporal networks and their randomized counterparts; which are used as null models in order to study as a term of comparison, to verify whether the temporal networks in the medium systems size display some non-trivial features. Our proposed measurement is based on the k-means clustering [24] and determines which samples to maintain and average in order to detect the impact of different correlations on “outbreak size” and “probability of the outbreak”. These samples would then represent the high-risk outbreaks.

Our studied empirical temporal networks, namely a school, a conference and a hospital network, are not large topologies in thermodynamic limit, however they are practically important for epidemic crisis management. The diamond princess ship is another good example of such cases in the COVID-19 pandemic crisis [25]. While for the networks in the thermodynamic limit, a simple ensemble average of the simulation realizations can be good enough to calculate the risk of epidemics, we will show that for these empirical networks, this is not the case and our proposed measurements can address this issue better.

## Materials and methods

### Topology: Empirical temporal networks

Here we study three different empirical temporal networks. All data sets are representative of close-range interactions among individuals, as captured by wearable sensors they carried. These data sets contain the list of contacts recorded within a specific time period. Every contact is characterized by the labels of the individuals interacting, as well as the time of their interaction. The data sets follow the “contact sequence” format, contrasting with the “interval graph” picture [26, 27]. The networks are:

1. *Hospital Network*: “Contacts between patients and health-care workers in a hospital ward in Lyon, France, from Monday, December 6, 2010 at 1:00pm to Friday, December 10, 2010 at 2:00 pm” [28].
2. *Conference Network*: “Face-to-face interactions between ACM Hypertext 2009 conference attendees” [29].
3. *Primary School Network*: “Contacts between children and teachers in a primary school in Lyon, France during two days in October 2009” [30, 31].

Some characteristics of these networks are summarized in Table 1.

**Table 1:** Some characteristics of the three different empirical temporal networks: Hospital network, conference network and primary school network. The details of the data collection and metadata can be found respectively in [28–30].

Data	Vertices #	Contacts #	Duration	Resolution	link/t Ave
Hospital	75	32424	4 days	20s	0.093
Conference	113	20818	3 days	20s	0.098
Primary School	242	125773	32 hours	20s	1.076

#### Shuffling temporal correlations

In order to study effects of different temporal correlations on any spreading phenomena, one has to shuffle the correlations. Each shuffling method randomizes some of the correlations and preserves the rest. A comparison of the spreading dynamics on the original and shuffled networks determines the impact of that specific temporal correlation on the dynamics.

We first introduce some attributes of our temporal networks:

1. D: Daily pattern: As it is shown in the first set of graphs, the daily patterns correspond to the frequency of the events, namely contact occurrences, could be described by the inter-event time distribution of the event.
2. C: Community structure: The characteristics of the aggregated counterpart of the network, such as clustering and clique formations.
3. B: Bursty event dynamics of single links: Burstiness of the time series of occurrences of single edges.
4. W: Weight-topology correlations: A weight is the number of occurrences of each edge. The weight-topology correlations comprise the characteristics of the statistics of the edges’ weights.
5. E: Event-event correlations between links: The correlations of occurrence between edges. These are characterized by the order of appearance of the edges.

For detailed descriptions of the above characteristics see [26].

Some of the shuffling methods we implemented are referenced in [17] by the network attributes they preserve. Furthermore, a comprehensive naming convention has been designed in [27] to label different methods of temporal network shufflings; we will introduce our shuffles, using both conventions.

We define the time-stamps at which every edge has appeared as the “single-link event sequence”, and the number of appearances of an edge as a weight.

**Fig 1:**
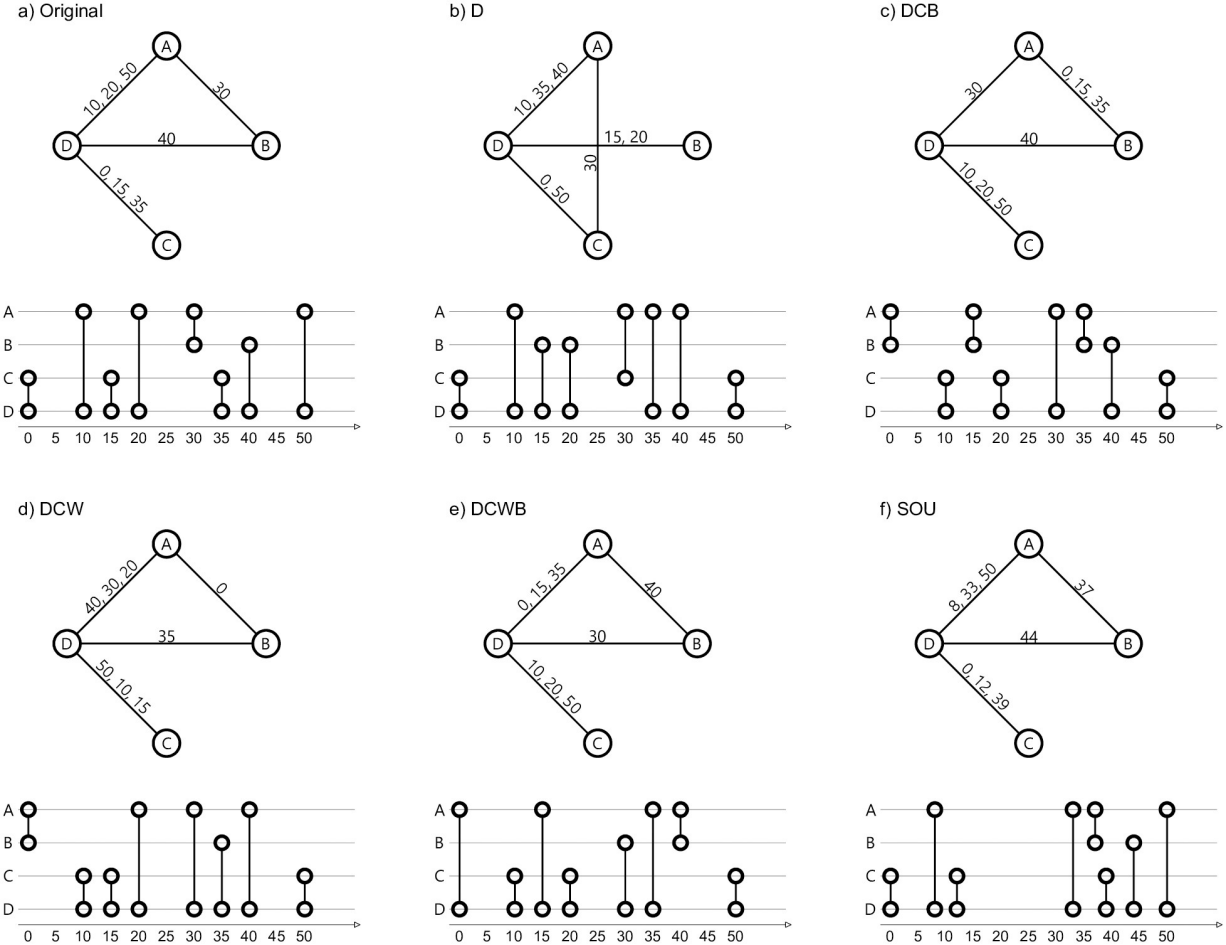
Visualization of a sample original network and its shuffled counterparts. For each network, the upper figure demonstrates the aggregated network with times of contacts as labels on edges. And the lower figure demonstrates a timeline for vertices, while the vertical line denotes the existence of a contact between two vertices at that specific time-step.

The shuffling methods, also demonstrated in Fig 1 are listed below:

1. DCWB (equal-weight link-sequence shuffled): “Whole single-link event sequences are randomly exchanged between links having the same number of events. Temporal correlations between links are destroyed” [17]. The process is represented as $P[w,p_{L}(\Theta )]$ in [27].
2. DCB (link-sequence shuffled): “Whole single-link event sequences are randomly exchanged between randomly chosen links. Event-event and weight-topology correlations are destroyed” [17]. The process is represented as $P[L,p_{L}(\Theta )]$ in [27].
3. DCW (time-shuffled): “Time stamps of the whole original event sequence are randomly reshuffled. Temporal correlations are destroyed” [17]. The process is represented as *P*[***w***, ***t***] in [27].
4. D (configuration model): “The original aggregated network is rewired according to the configuration model, where the degree distribution of the nodes and their connections are maintained but the topology is uncorrelated. Then, the original single-link event sequences are randomly placed on the links, and time shuffling as above is performed. All correlations except seasonalities like the daily cycle are destroyed” [17]. The process is represented as $P[k,I_{\lambda },p\left(w\right),t]$ in [27]. As we retain the original time-stamps, daily patterns are preserved. Since this kind of shuffling may destroy the connectivity of the network, the dynamics should be instead simulated on the giant cluster of the shuffled network. Although in our case the giant cluster remains intact.
5. SOU (same-ordered): We introduce a new method of shuffling for temporal networks. Using a uniform random distribution, we create a new time-series for our temporal network, and we assign the new time-series to the occurrence time of our contacts, while preserving the ordering of the events. Unlike the previously implemented shuffles, the ordering of the events (appearance of the edges) will remain intact, but the time difference correlations (daily patterns) will be destroyed.

Please note that all of the three original aggregated networks have a single connected component, which is as large as the system size. Also, all of the shuffling methods have preserved the size of the connected component for the aggregated shuffled network.

### Dynamics

**Fig 2:**
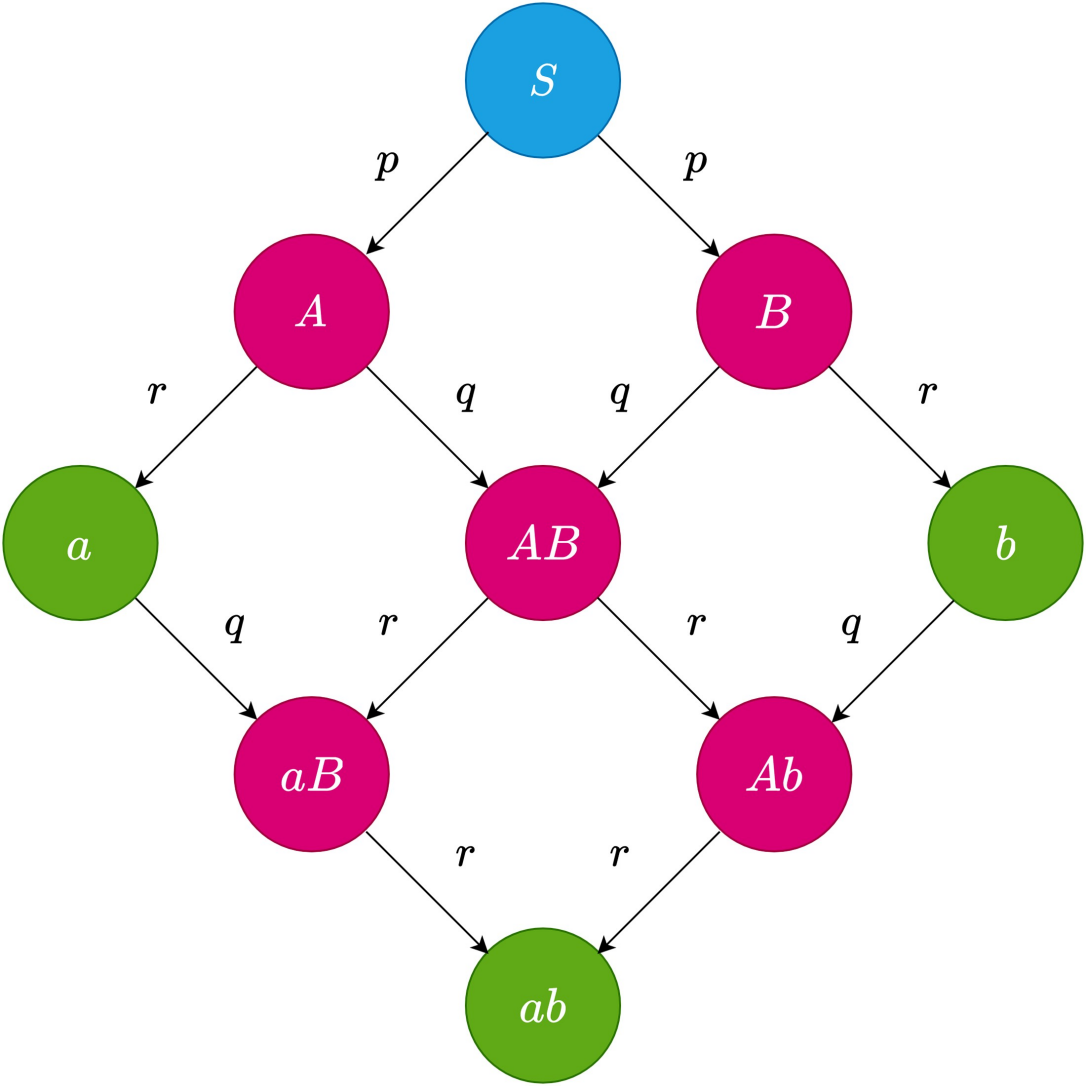
Different states in the SIR-SIR model [18] and the probabilities of switching between states. Capital letters denote the infectious state regarding one disease, small case letters denote the recovered states. Each arrow indicates a transition with a certain probability. State S illustrated by color blue, denotes the agents’ initial states. Blocks illustrated by color pink and green, respectively represent infectious and recovered states.

In this model each agent can be in one of the three different states: “S-I-R” (Susceptible-Infected-Recovered) based on its status regarding the two diseases. This in total makes up 9 different states which are displayed in Fig 2. The simulations are conducted using rejection-based modeling [32]. The simulation steps consist of the following:

1. Every infectious agent, turns its susceptible neighbors to infectious for the same disease with infection probability *p*.
2. Every infectious agent, infects its neighbors, which have already been infected (or recovered) by the other disease, with the secondary infection probability *q*.
3. Every infectious agent recovers from each disease with recovery probability *r* [33].

**Fig 3:**
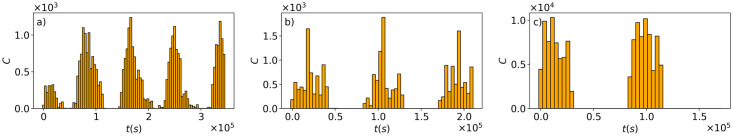
The periodic behavior of the number of contacts for 3600s aggregated time intervals for the a) Hospital Network, b) Conference Network and c) Primary School Network.

The two diseases generally act independently, except for when an agent has been infected with one of the diseases (regardless of whether it is recovered or still infectious), where the probability for becoming infected by the other disease is *q*. The three parameters *p*, *q* and *r* are our control parameters. Since the number of control parameters is relatively high, we set a specific value for *r*, for each temporal network, considering the frequency and distribution of its contacts, as seen in Fig 3 and Table 1. As in [16] we study the inactivity periods within each activity histogram. To examine the effects of each network’s dynamics, we need to consider a low enough recovery rate to enable the infection to survive during the inactivity period. On the other hand, considering a very low recovery rate will decrease the speed of the spreading dynamic. It has been reported that, when the dynamics on the network is much slower than the dynamics of the network itself, the temporal aspects of the network can be neglected and the results will be the same as those on the weighted aggregated counterpart of the network [34]. Therefore, to capture temporal effects, we consider the length of the valleys as an indicator for a suitable recovery rate, as depicted in Fig 3. For that, we consider the time-scale of the valleys for both the hospital and the conference networks which is approximately the size of 1000 steps (each step is 20 seconds), and assign *r* = 0.001. In the case of the primary school, the size of the valley is of the order of 5000 time steps (each step is 20 seconds), so we assign *r* = 0.0002. We also consider two cases for *q*, called cooperative and independent spreading as indicated in [16]. In the former situation, we set *q* = 1 so that the probability of acquiring a second disease would be higher than the first one (the range of values for *p* are drastically lower than 1). And in the latter, we set *q* = *p*, so that the spreading would be totally independent.

The simulation runs until it reaches the stationary state, i.e., until all of the agents reach one of the blue or green states of Fig 2. A temporal-periodical boundary condition is applied to the contact networks, in other words, we loop over the contacts, until the diseases die off.

The initial condition is set to a single randomly chosen doubly infected node.

### Macroscopic observables (order parameters)

**Fig 4:**
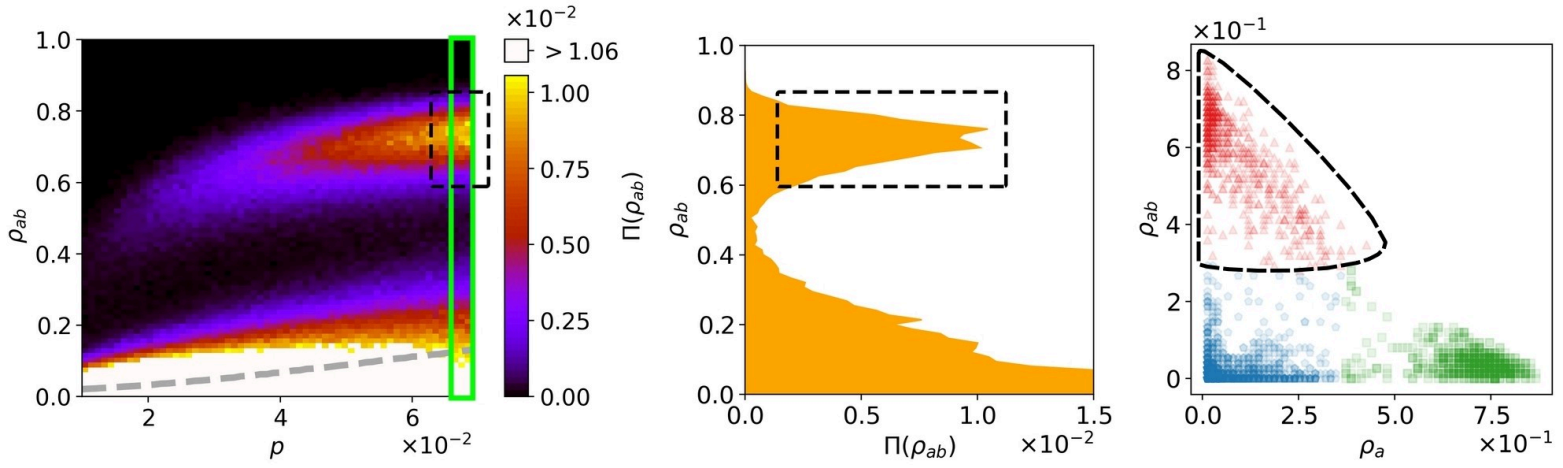
Systematic measurement of *P*_*ab*_ (Eq 1) and $\bar{ab}$ (Eq 2). The figure demonstrates how the k-means clustering measure works. As an example, we see the coinfection simulation on the DCWB shuffled hospital network with *q* = 1 and *r* = 0.001. The left panel shows the density of the *ab* population, and the probability that a realization settles on the given value of the density (*π*(*ρ*_*ab*_): color axis) while varying p, which is the first infection probability. The gray dashed curve demonstrates the average *ρ*_*ab*_. The middle panel distinguishes precisely the two epidemic branches in the left panel at *p* = 0.069, the vertical window. The right panel shows the fraction of individuals recovered from one disease (x axis) and from both diseases (y axis), within each realization. Each marker denotes a single realization and different colors and shapes indicate different clusters: red triangles (double infection outbreaks), green squares (single infection), blue pentagons (no outbreaks). The dashed shapes, encircle the realizations which make up the outbreak branch (OB) in each illustration. 50000 realizations have been simulated, but for illustrative purposes, only a sample of 5000 realizations are depicted in the right panel.

With the aforementioned dynamics, each run can be described by *ρ*_*i*_ the fraction of nodes in the *i* state, at the equilibrium. The most common macroscopic observable is the ensemble average fraction of the doubly recovered individuals, which in this case is 〈*ρ*_*ab*_〉. However, as shown in [16, 19] due to the branching effect of the coinfection dynamics, the average would not be a good indicator of such epidemic behaviors (see also Fig 4). Moreover, this may not be specific to the situation of coinfection and due to finite size effect one can observe branches with somewhat similar results, also for independent spreading. The histograms in the left panel of Fig 4, and in the S1 Appendix. Simulation result heat maps show at least two branches: one formed around the *ρ*_*ab*_ = 0 and the other, namely the outbreak branch, formed around a higher value. The outbreak branch represents the high-risk outbreak instances. Therefore, instead of averaging the whole distribution, we only average over the outbreak branch. In particular, we take the mean outbreak size ($\bar{ab}$), and moreover look at another order parameter, namely the outbreak probability (*P*_*ab*_), the probability for a realization to land on the outbreak branch. In simple terms, the first parameter indicates the pervasiveness of an outbreak, and the second implies how likely it is that an outbreak would occur. These parameters are mathematically defined in Eqs 1 and 2, where *ρ*_*ab*_ is the fraction of doubly recovered agents, *π*(*ρ*_*ab*_(p*)) is the distribution of *ρ*_*ab*_ for an arbitrary p* value of the control parameter and the integration over the outbreak branch (*OB*), accounting for high risk outbreaks.

$$\begin{array}{c} P_{ab}\left(p^{*}\right)=\int_{OB}^{}\pi (\rho_{ab}\left(p^{*}\right))d\rho_{ab}\left(p^{*}\right) \end{array}$$ (1)

$$\begin{array}{c} \bar{ab}\left(p^{*}\right)=\int_{OB}^{}\pi (\rho_{ab}\left(p^{*}\right))\rho_{ab}\left(p^{*}\right)d\rho_{ab}\left(p^{*}\right) \end{array}$$ (2)

The main two branches are not always well distinguishable, especially in the case of coinfections [19]. Therefore, we introduce a new method, using k-means clustering [24, 35] to determine their values.

### k-means clustering

Simulation realizations evolve according to one of the following scenarios: 1. No outbreak: There is no considerable growth for either of the diseases. The dynamics dies out early in the process and settle on the lower branch in Fig 4, left and middle panels. 2. Single outbreak: One of the diseases dies out early and the other grows considerably independently. This means that cooperation does not affect the process. Similarly, dynamics lands on the lower branch. 3. Mutual outbreak: Both diseases grow considerably. This type of dynamics settles on the upper branch in Fig 4, left and middle panels.

Taking the above into account, to address the issue of distinguishing the outbreak branch from the lower branch, in addition to the *ab* recovered ones, we also look at the *a* (or *b* as the dynamics is symmetrical) recovered ones. In the right panel of Fig 4, these scenarios are depicted in 3 clusters, blue (pentagons), green (squares) and red (triangles) respectively. The k-means clustering method provides us with a systematic measure to classify these cases and find the red cluster in the right panel, as a counterpart of the upper branch in the left and middle panels. The k-means clustering is an unsupervised method of partitioning data into a specific number of clusters so that the data points within the same cluster will have the minimal distance from each other, as compared to the data points of other clusters [36]. Here *ρ*_*a*_ and *ρ*_*ab*_ are the parameters employed to devise an Euclidean distance between the data points. The only input this algorithm requires is the number of desired clusters. Finding the optimal number of clusters is an important task and several methods have been introduced to address this issue [37]. However, since we have an argument and intuition about the number of branches/clusters in our data set: mutual outbreak, single outbreak and no outbreak, we are able to proceed with this algorithm considering only 3 clusters.

The k-means algorithm, its description and also an illustration of its procedure are provided in S2 Appendix. k-means algorithm and illustration.

By defining the red cluster which corresponds to the outbreak branch, we can now quantitatively define *P*_*ab*_ as the fraction of realizations which fall in the outbreak branch and $\bar{ab}$ as the outbreak branch center.

## Results

We proceed to illustrate the phase space of *p*, so as to observe the behavior of the fraction of doubly recovered agents (*ρ*_*ab*_). We first derive the distribution of the *ρ*_*ab*_ for each set of parameters and networks. To visualize our results, we use 2D histograms consisting of 1D histograms for each *p* value, as shown in Fig 4, left panel and also in the S1 Appendix. Simulation result heat maps. The first point we observe by investigating these heatmaps is the change in *p*_*c*_ or the outbreak threshold. This parameter which indicates the lowest value of *p* that causes a significant outbreak, is lower for all coinfection spreadings (*q* = 1), compared to their independent (*q* = *p*) spreading counterparts.

**Fig 5:**
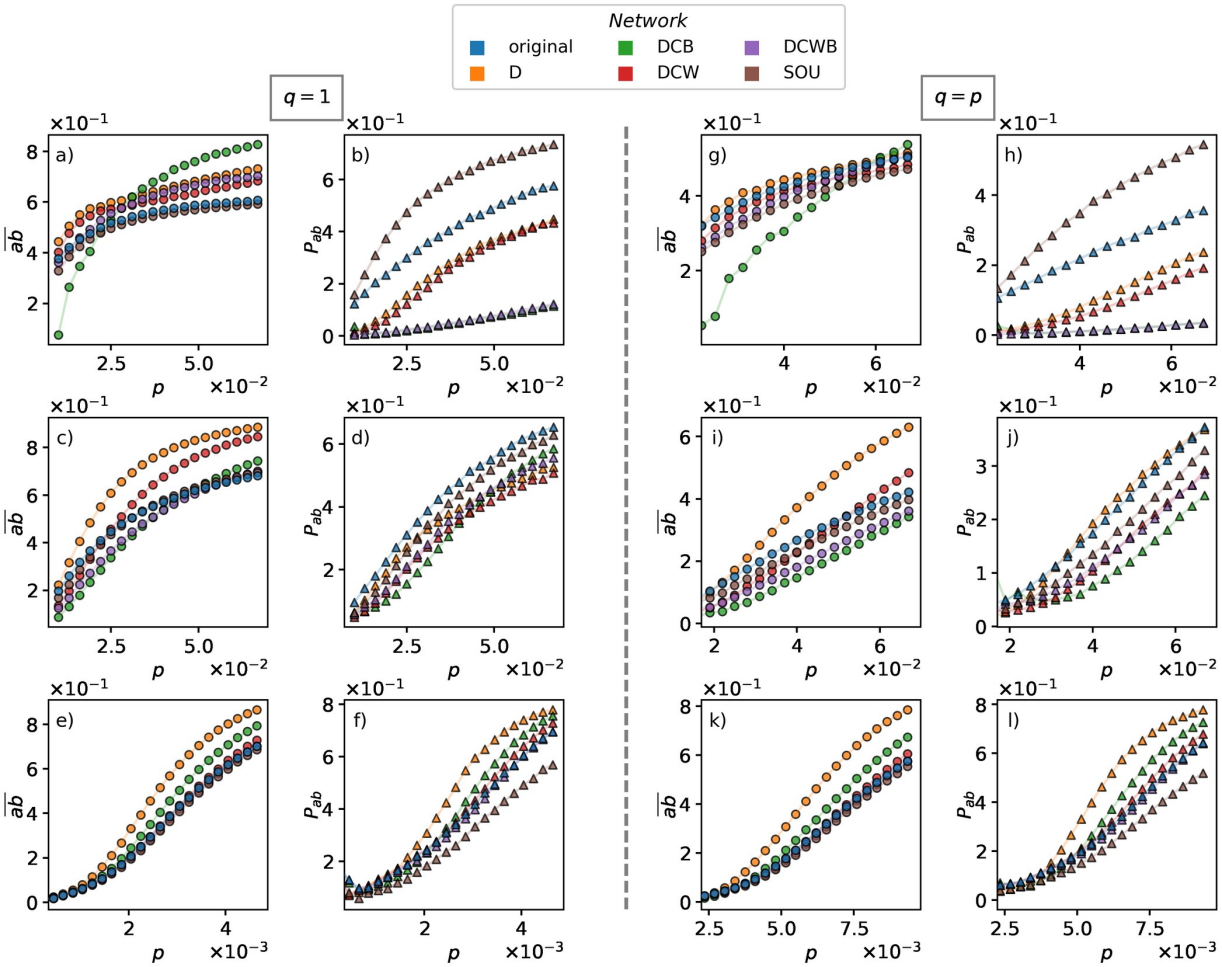
Mean of the outbreak size ($\bar{ab}$), columns 1 and 3, and the outbreak probability (*P*_*ab*_), columns 2 and 4, for coinfective SIR-SIR (*q* = 1), columns 1 and 2, and independent SIR-SIR dynamics (*q* = *p*), columns 3 and 4. First, second and third rows shows results for hospital, conference and primary school networks respectively. These values are respectively obtained based on the introduced method in the Fig 4 right panel, i.e., the fraction and average *ρ*_*ab*_ of red triangles in the right panel.

Furthermore, we calculate *P*_*ab*_ and $\bar{ab}$ for different shuffled versions of our networks in Fig 5.

As mentioned, all of the shufflings starting with capital D (we will call them D family shuffles), retain the daily patterns i.e., original time-series of the network, while destroying the ordering of the events, (event-event correlations), and therefore the causality between the events. On the other hand SOU shuffling retains the event-event correlations and randomizes the time-series.

We firstly summarize our observations for **coinfection** scenario in Fig 5 panels a-f:

1. D family shuffles will increase the value of $\bar{ab}$ for high *p* values, therefore in case an outbreak happens it would be more hazardous. This may be due to the fact that in the empirical networks with an underlying spatio-temporal structure, there exists a localized behavior, meaning that individuals will interact with the same group of people instead of exploring new people. Therefore, they keep the disease in their proximity during short periods of time. Additionally, spatio-temporal structures will raise transitive relations, so the individual will have stronger clustering compared to randomized networks. For example, if an individual “Sina” is interacting with another individual “Fakhteh”, and “Fakhteh” is interacting with “Reza”, then the probability that “Sina” and “Reza” interact with each other in a short period of time, is higher than the average probability of interaction between two individuals. This is due to both spatio-temporal features of the network and the social relations among individuals. By destroying the event-event correlations the individuals will have a higher chance to interact with more people during a short period, therefore leading to an increase in the size of a possible outbreak. Due to the stronger clustering in the original networks, the pathogens can be trapped in a spatio-temporal structure. Since D family shuffling methods randomize these clusters, the trapping probability gets reduced and the two diseases interact more often. Thus, if they could meet, the fraction of doubly recovered people would increase.
2. SOU shuffle does not have any strong impact on $\bar{ab}$. This agrees with the results in [38], although only a single SIR dynamics is studied in their case. Holme and Liljeros have performed a type of shuffling named Inter-event Interval Neutralized (IIN) method, which sets a uniform time distribution to the activity of each edge, within its original first (birth) and last (death) appearance. By considering the average number of recovered individuals during SIR spreading, the authors concluded that inter-event time distributions do not have a strong impact on the spreading, while the times of birth and death of a link are important. One should note that, the SOU shuffling keeps the order of the events and redistributes the time intervals between all events while the IIN method [38] redistributes the time intervals between the events of the same type. This means that IIN may lead to reordering of the events in small time durations. However, these methods are of the same nature since they both keep intact the order of appearance of the edges and the activity clock [39] between birth and dead of a link, while destroying the inter-event time distributions.
  Nevertheless, as we can observe in Fig 5, panels b, d and f, SOU shuffling can either decrease or increase the outbreak probability (*P*_*ab*_). This means that the inter-event time distributions may affect the dynamics but we would need to look at the proper order parameters and averaging to notice their effect. For instance, Holme and Liljeros have only studied the outbreak size ($\bar{ab}$) averaged over all realizations and concluded that inter-event time distributions have no strong impact on the dynamics.
3. Some correlations which hinder the process of coinfection for a range of control parameters, may also enhance the spreading for another set of parameters. Panels a and c of Fig 5 present such examples, where the DCB shuffles have a smaller $\bar{ab}$ in comparison to the original networks for *p* < 0.03 (hospital) and *p* < 0.05 (conference). Nevertheless, for the DCB shuffling, the $\bar{ab}$ manages to surpass the value of the original network for greater values of the control parameter. Hence it should be noted that the effect of link weight correlations on spreading phenomena is highly dependent on the range of the control parameter.
  Some recent studies discussed that temporal correlations can either facilitate the spreading dynamics or weaken such processes [34]. Here, our results show that both scenarios can happen depending on where the system is in the parameter space, which represents the dynamical and topological characteristic-times of the system.

Secondly, we compare the results of coinfection (*q* = 1) in Fig 5 panels a-f, with independent spreading (*q* = *p*) in Fig 5 panels g-l. We observe that:

1. The D family shufflings do not show any consistent effect on $\bar{ab}$, while they increase this value for coinfections. This signifies the order of the events and also spatio-temporal correlations having a more intensive effect on coinfection compared to the independent spreading dynamics. This could be caused by the fact that the formation of the spatio-temporal communities may constraint the spreading of each infection in separate communities. Considering that a collision of the two diseases leads to a greater effect on the $\bar{ab}$ for the coinfection, this type of correlations has a greater effect on the coinfective dynamics. In other words, neighboring effects caused by the existence of smaller communities in temporal networks, decrease the chance of two diseases interacting with each other. So, the lack of interaction between the two diseases for a proper time can have a stronger impact on the coinfection dynamics compared to the independent dynamics.
2. SOU shuffle does not have a strong effect on $\bar{ab}$, though it can affect (increase or decrease) *P*_*ab*_, which is the same as the case of coinfection.
3. Similar to the coinfection cases, Fig 5g shows that some correlations which hinder the process of spreading for a range of values of the parameter *p*, may also enhance it in other ranges.
4. Both order parameters $\bar{ab}$ and *P*_*ab*_ are smaller in comparison to the coinfection case (*q* = 1).

**Fig 6:**
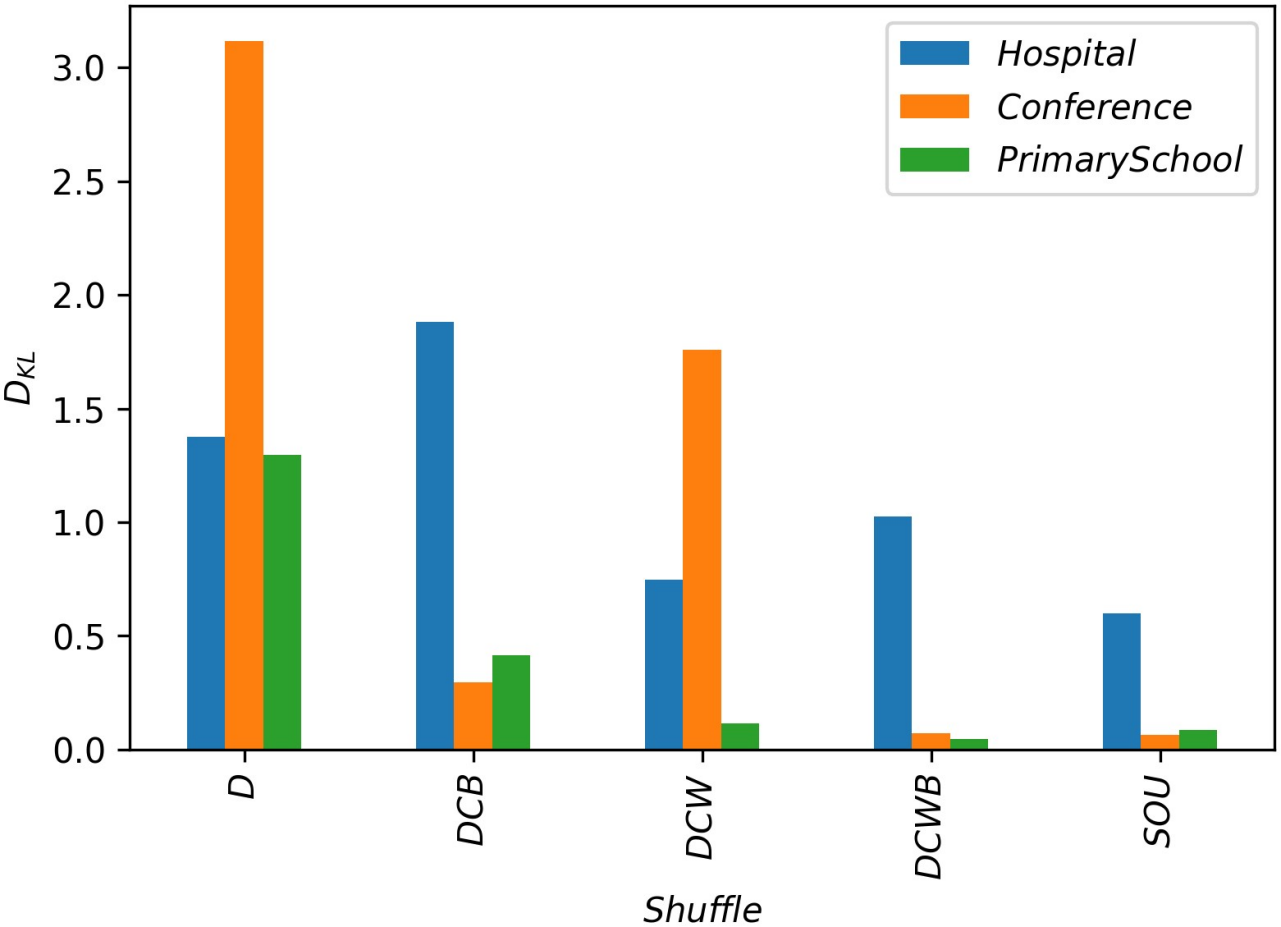
The value of cross-entropy for the dynamic results under each shuffle in relation to the original network for the value: *p* = 0.07 for hospital and conference networks and *p* = 0.005 for the primary school network. This parameter indicates the difference between the result of *ρ*_*ab*_ distribution for coinfection (*q* = 1) on each shuffled network and its original counterpart.

Finally, as an alternative comparative approach, we can quantitatively compare the results of the dynamics under different shufflings. To this end, we compute the cross-entropy or Kullback–Leibler divergence [40] for the probability distribution of *ρ*_*ab*_ for each shuffled network in relation to the original network. To avoid the singularity caused by the logarithm in the cross-entropy formula we increase the values of the histogram by the minimum non-zero value and then normalize the probability distribution again. The values for the cross-entropy in relation to the original network, for our shuffled networks can be seen in Fig 6. We observe that generally the dynamics has the closest behavior on the SOU shuffled networks in comparison to the original networks. Since the SOU shuffling keeps the order of events intact, we can conclude that for such processes the sequence of events has the strongest effect on the results. Furthermore, we show that although the Kullback–Leibler divergence is taking into account the whole *ρ*_*ab*_ distribution (Fig 4 middle panel) in comparison to the k-means clustering which focuses only on the outbreak branch (Fig 4 red triangles in the right panel), the two measures agree that the D shuffling has the greatest impact on the conference and the primary school networks of Fig 5 panels c, e, while DCB has the greatest impact on the hospital network, see in Fig 5 panel a.

It should be noted that SOU, DCW and DCWB shuffles retain the topology of the aggregated static counterpart of the original network and only randomize the edge list with respect to the time, on the other hand, D and DCB shuffles randomize this underlying static network. DCB shares the same edges with the original network but shuffles the edge weights (number of occurrences), while D shuffle only preserves the node degrees and even randomizes the edges of the static aggregated network. Both the k-means and the cross-entropy analyses indicate that D and DCB shuffles have stronger effects on the epidemic process, signifying the importance of the static topological correlations. In other words, while randomizing the temporal correlations of the network, namely the timing (SOU) and ordering (DCW-DCWB) has strong effect on the spreading results, randomizing the weight topology correlations (DCB) or the static topology itself (D) is more effective in altering the dynamics.

## Discussion

In summary, in this work, we studied the effects of various temporal correlations on the spreading process, both considering independent infection and coinfection. The processes were of the SIR type and simulated on three different temporal empirical networks, as well as on several of their shuffled counterparts. Each of the shuffling methods preserved some temporal correlations and randomized others. For instance, we introduced SOU shuffling which keeps the ordering of the events intact while randomizing the frequency of the events. We argued that in order to see the impact of any temporal correlation we need to investigate properly two order parameters: the probability of outbreak (*P*_*ab*_) and the outbreak size ($\bar{ab}$) as macroscopic observables. Moreover, since in many cases a simple ensemble averaging may lead to misinterpretation, we introduced a systematic measurement to identify the proper samples for calculating these two order parameters, which represent the high-risk outbreaks. This measurement is based on the k-means clustering method. Furthermore, we introduced an alternative method, namely the Kullback–Leibler divergence which calculates the difference between the distributions of *ρ*_*ab*_ for the shuffled and original networks. While k-means clustering identifies the proper samples and then takes an average over them, the Kullback–Leibler divergence computes the discrepancy between the two histograms cell by cell, and then takes an average. We showed that both measurements agree on which shuffling has the dominant impact on each network. By categorizing the shufflings into the ones that preserve the static aggregated network’s topology (SOU-DCW-DCWB) and those that randomize it (D-DCB) and comparing the results via both the k-means and cross-entropy methods, we conclude that while merely temporal shufflings strongly affect the spreading process, randomization of the static topology has the most drastic effect.

We observed that cooperation between two diseases facilitates the spreading dynamics on original networks as well as on shuffled networks. In the coinfection process, randomization of the sequence of the events makes the outbreak more pervasive, i.e., $\bar{ab}$ increases. On the other hand, these correlations do not have a consistent effect on the independent infection dynamics, and can either decrease or increase the outbreak size. This point indicates that the ordering of the events and the spatio-temporal features have a greater effect on coinfection. In both independent infection and coinfection, daily patterns have no strong impact on the outbreak size, while they can change the probability of outbreaks for various networks. Last but not the least, in order to understand the impact of temporal correlations on spreading phenomena, not only the proper order parameter and averaging matters, but also the dependency on the range of the control parameter.

Our proposed systematic measurement provides a more precise method to trace the macroscopic observables. Thus, it can help us to improve the epidemic risk calculations [16]. Also, our results can help organizers and managers better organize gatherings, in order to decrease high risk outbreaks. For instance, some sort of shuffling of the shift charts in a hospital can decrease the probability of a high-risk outbreak.

This method not only improves our understanding of the dynamics “on” the networks, but can also open the road to better understand the topological features of temporal empirical networks, touching the topic of the dynamics “of” the networks.

Nevertheless there are some limitations: firstly, the collected data represents the temporal interactions between individuals only in the range of one and a half days to four days. To overcome the time scale limitations we have used the temporal-periodical boundary condition. In other words, it has been assumed that the main topological features (Daily patterns—Weight-topology—Community structure—Burstiness—Event-Event Correlations) of such organizations, namely schools, hospitals and conferences would be conserved over longer periods. In cases where the characteristics of the network evolve over time and/or long-term correlations are prevalent, more data gathering would be necessary. Secondly, our results and conclusions are restricted to the SIR type dynamics, therefore other spreading dynamics including SIS type dynamics as well as dynamics with significant latent periods need to be investigated in future studies. Thirdly, here we studied only an extreme scenario, namely the strong cooperation (*q* = 1) in comparison to the independent dynamics (*q* = *p*). Weaker cooperation (*p* < *q* < 1) as well as competition (*q* < *p*) scenarios may have different behaviors and should be studied more precisely in future works.

## Supporting information

S1 Appendix Simulation result heat maps. Illustration of the results of the spreading simulations for all networks and their shuffled counterparts for both independent SIR-SIR infection (**q** = **p**) and coinfective SIR-SIR (**q** = **1**) cases.
(PDF)

S2 Appendix k-means algorithm and illustration. (PDF)
