# Supplementary material for: Impact of temporal correlations on high risk outbreaks of independent and cooperative SIR dynamics
Source: PLoS One. 2021 Jul 20;16(7):e0253563. doi: 10.1371/journal.pone.0253563 (PMC8291698; doi:10.1371/journal.pone.0253563)
Supplement: S1 Appendix — Illustration of the results of the spreading simulations for all networks and their shuffled counterparts for both independent SIR-SIR infection (q = p) and coinfective SIR-SIR (q = 1) cases. (PDF) [file pone.0253563.s001.pdf]

S1 Appendix. Simulation result heat maps

In Figures 1, 2, 3, 4, 5, 6 we have illustrated the results of the spreading simulations for all networks and their shuffled counterparts for both independent SIR-SIR infection ($\mathbf{q} = \mathbf{p}$) and coinfective SIR-SIR ($\mathbf{q} = \mathbf{1}$) cases.

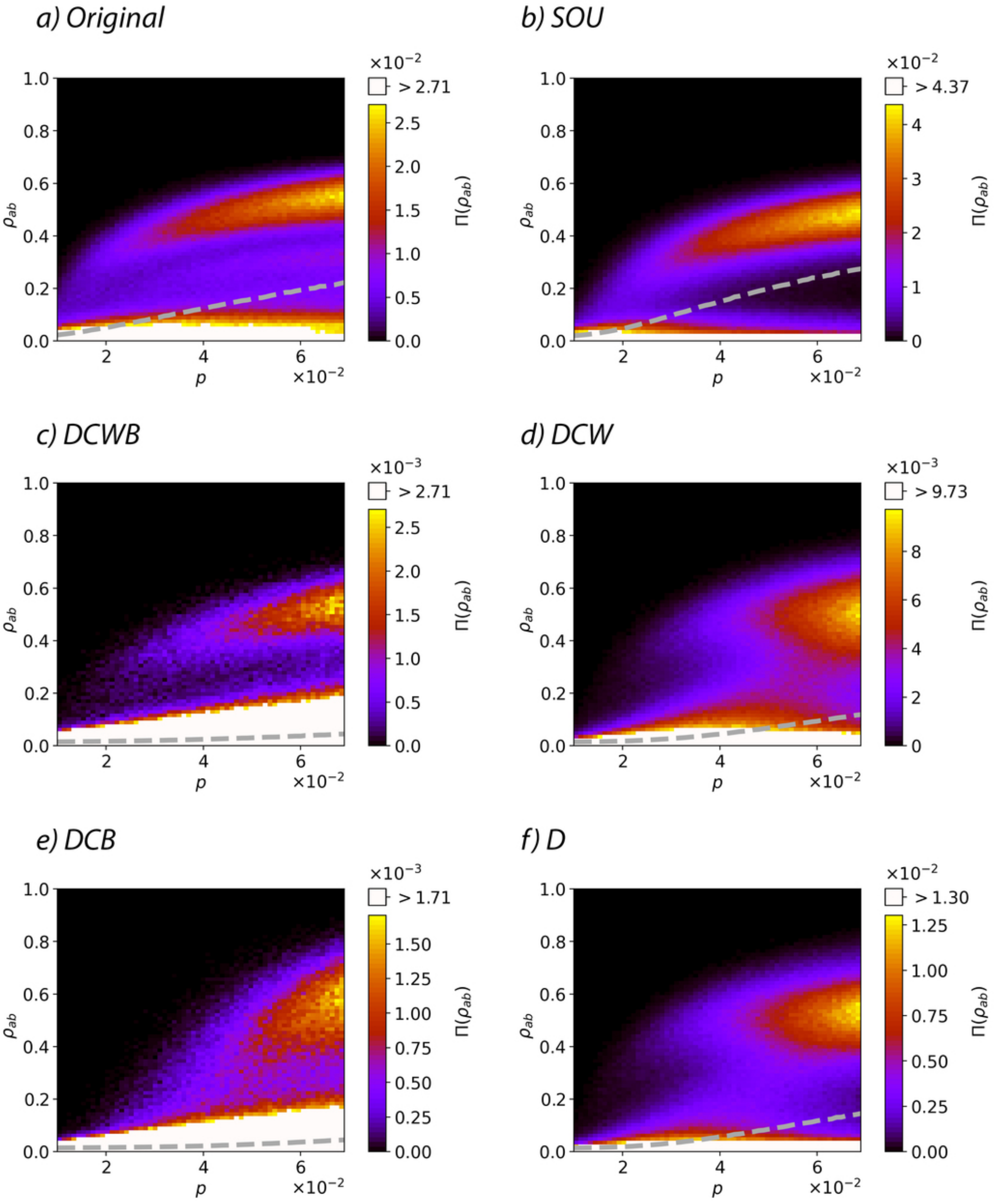

FIG. 1: The results of **independent SIR-SIR infection ($\mathbf{q} = \mathbf{p}$) simulation on the hospital network**, the x axis is the control parameter p, the y axis is the size of the final doubly recovered agents (ab), and the color axis denotes the fraction of realization with the specific value of ab, The gray dashed curve demonstrates the average ρ_{ab}. Please note that the coloring scale is different on each graph, to clarify the discrepancy between different regions of each graph.

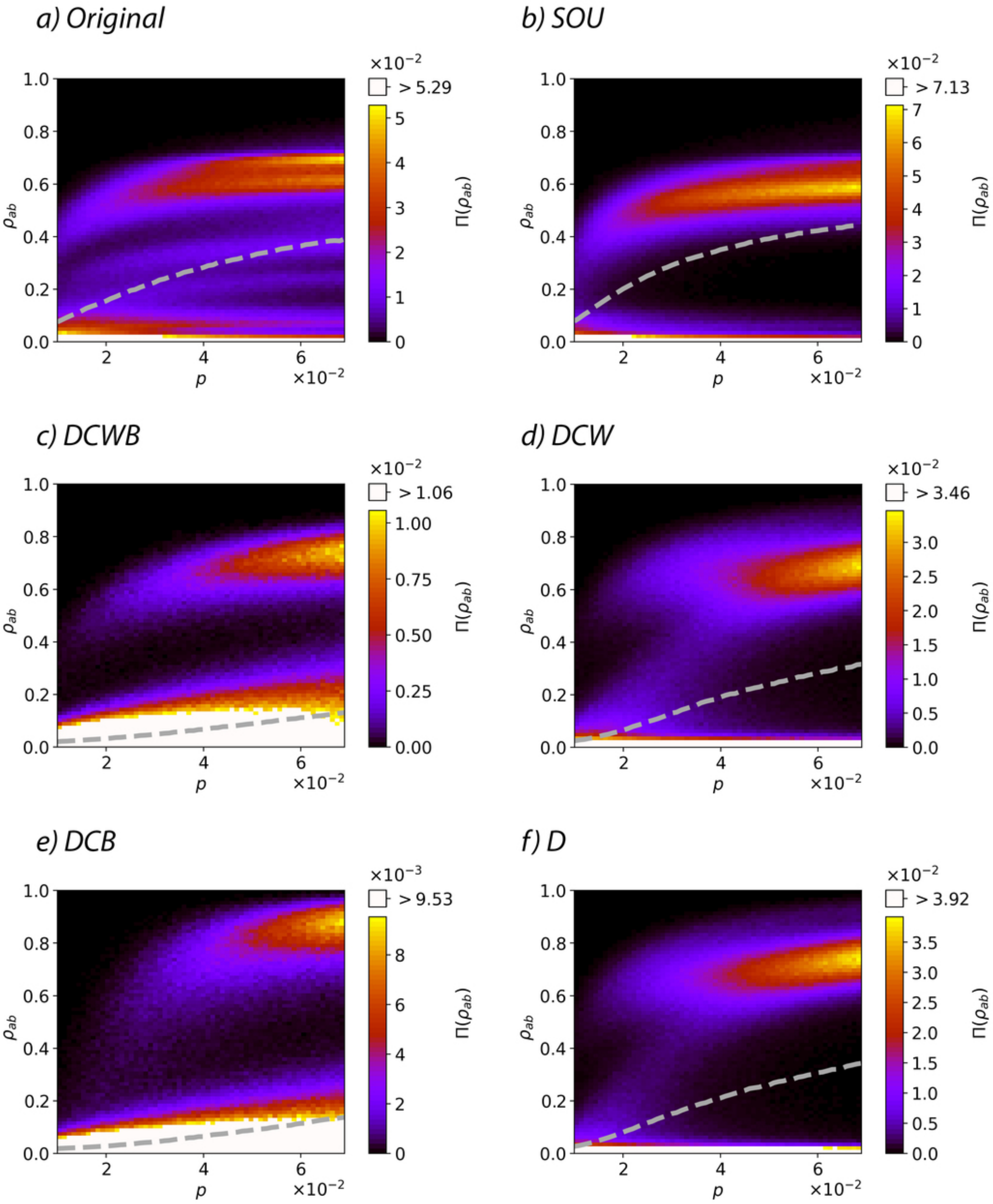

FIG. 2: The results of **coinfective SIR-SIR (q = 1) simulation on the hospital network**, the x axis is the control parameter p, the y axis is the size of the final doubly recovered agents (ab), and the color code denotes the fraction of realization with the specific value of ab, The gray dashed curve demonstrates the average ρ_{ab}. Please note that the coloring scale is different on each graph, to clarify the discrepancy between different regions of each graph.

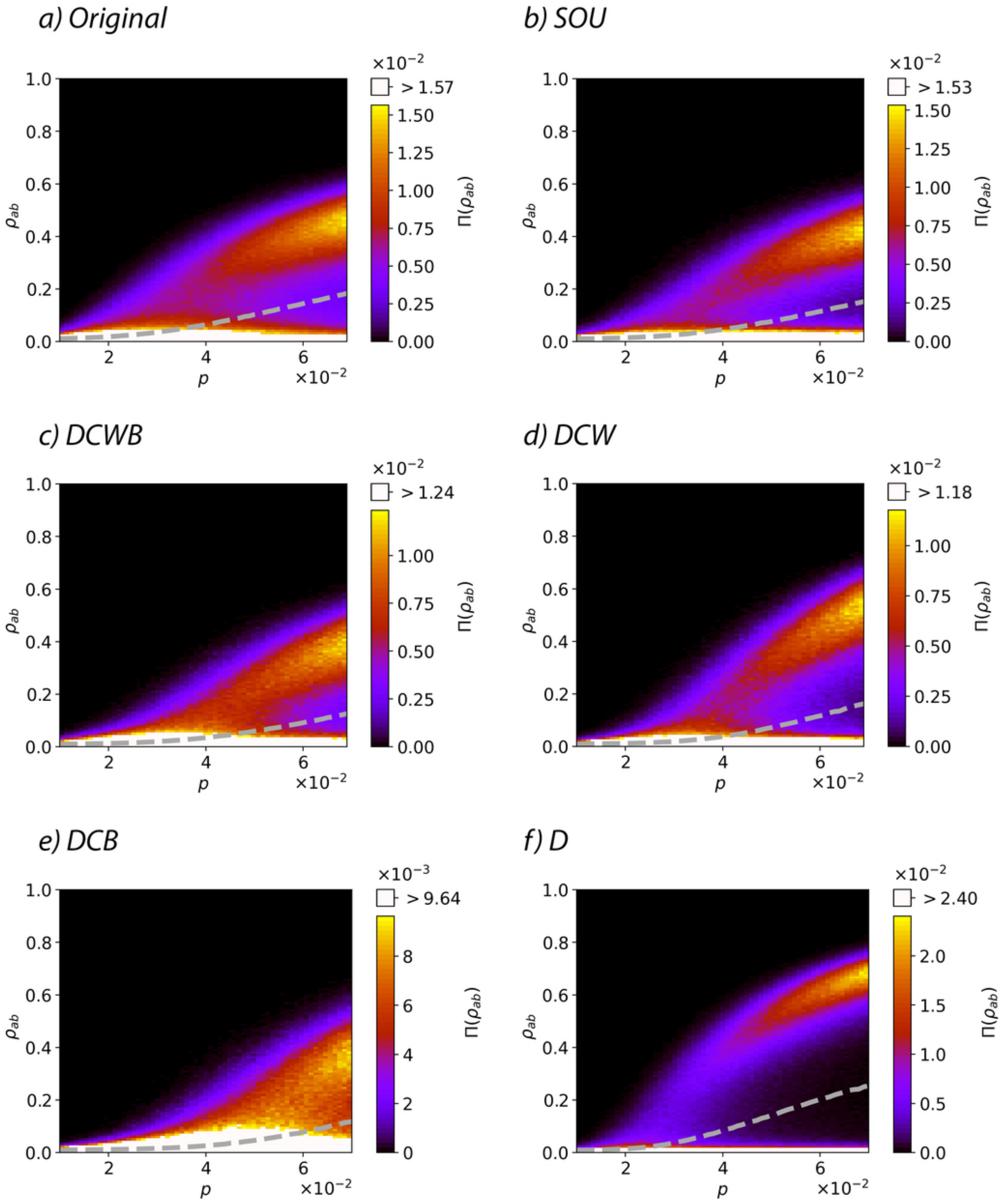

FIG. 3: The results of **independent SIR-SIR infection ($\mathbf{q = p}$) simulation on the conference network**, the x axis is the control parameter p, the y axis is the size of the final doubly recovered agents (ab), and the color code denotes the fraction of realization with the specific value of ab, The gray dashed curve demonstrates the average ρ_{ab}. Please note that the coloring scale is different on each graph, to clarify the discrepancy between different regions of each graph.

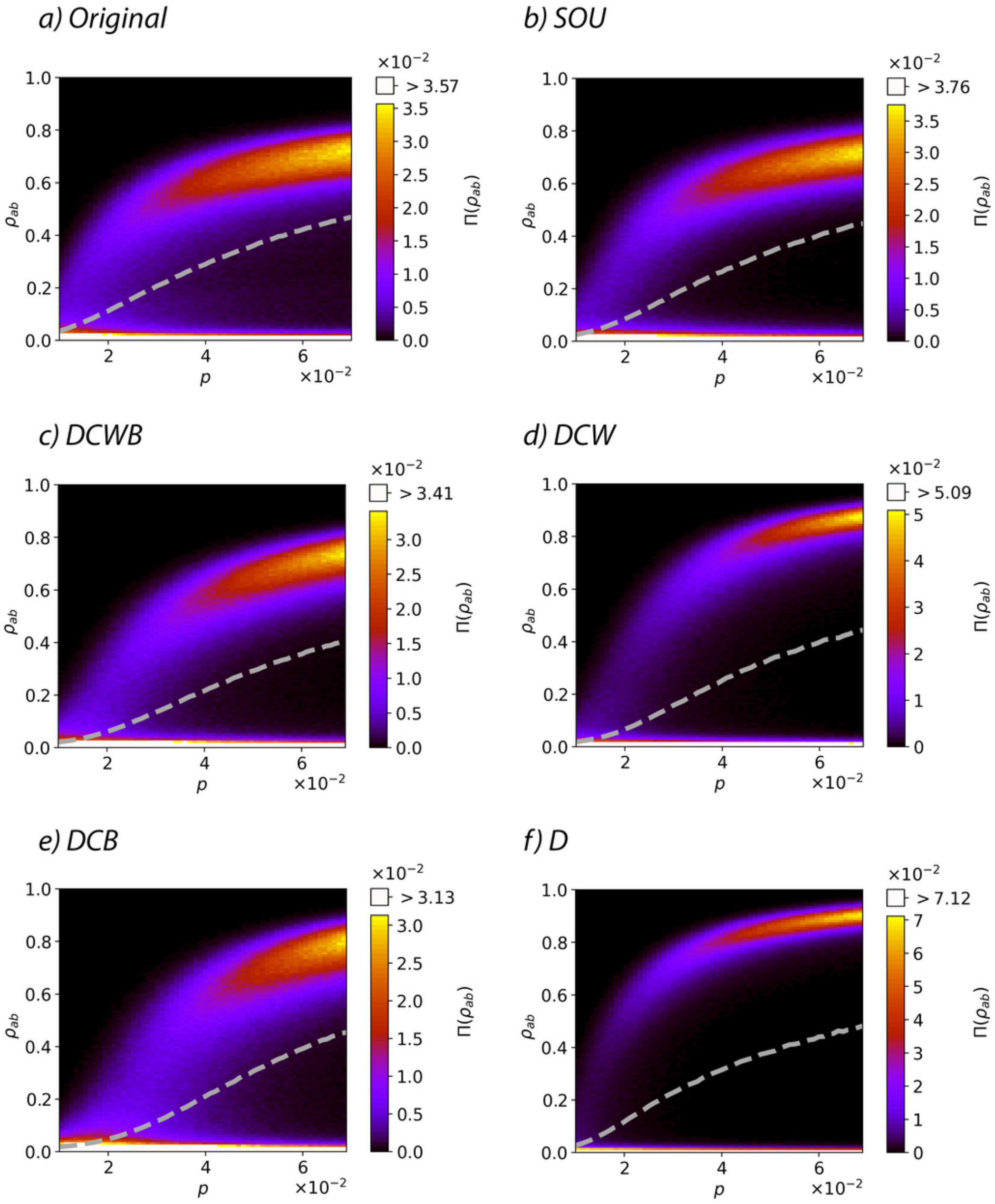

FIG. 4: The results of **coinfective SIR-SIR ($\mathbf{q = 1}$) simulation on the conference network**, the x axis is the control parameter p, the y axis is the size of the final doubly recovered agents (ab), and the color code denotes the fraction of realization with the specific value of ab, The gray dashed curve demonstrates the average ρ_{ab}. Please note that the coloring scale is different on each graph, to clarify the discrepancy between different regions of each graph.

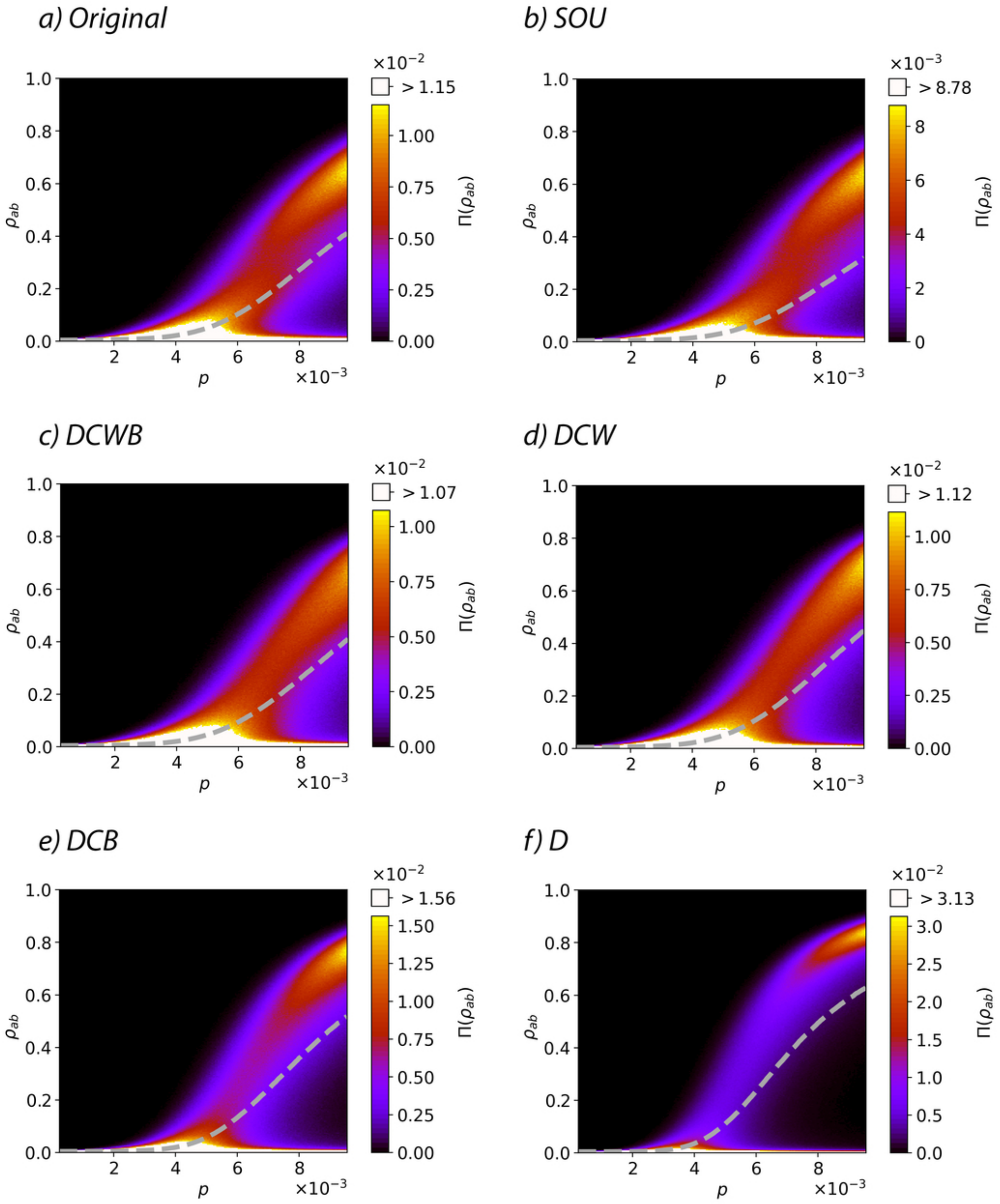

FIG. 5: The results of **independent SIR-SIR infection (q = p) simulation on the primary school network**, the x axis is the control parameter p, the y axis is the size of the final doubly recovered agents (ab), and the color code denotes the fraction of realization with the specific value of ab, The gray dashed curve demonstrates the average ρ_{ab}. Please note that the coloring scale is different on each graph, to clarify the discrepancy between different regions of each graph.

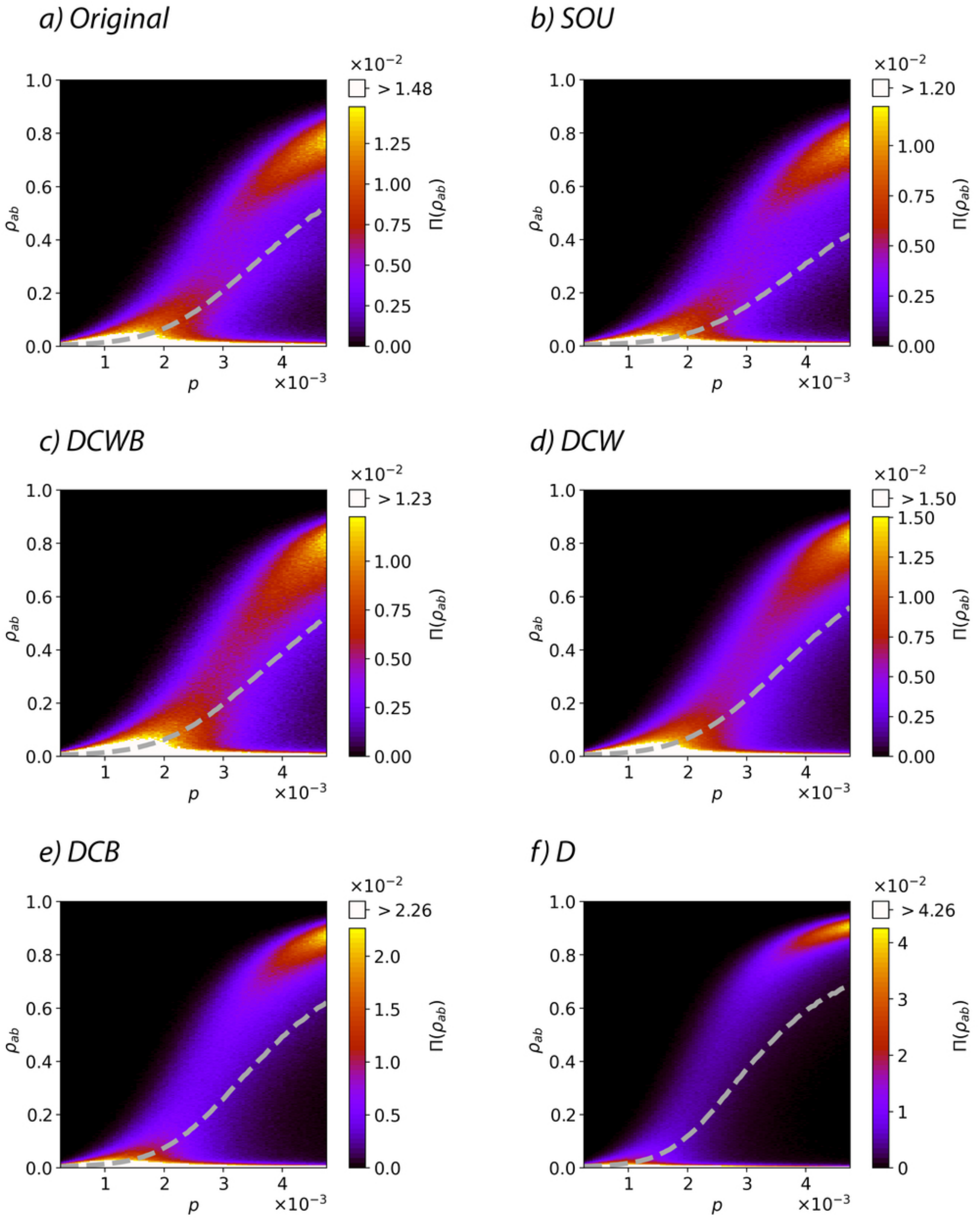

FIG. 6: The results of **coinfective SIR-SIR ($\mathbf{q = 1}$) simulation on the primary school network**, the x axis is the control parameter p, the y axis is the size of the final doubly recovered agents (ab), and the color code denotes the fraction of realization with the specific value of ab, The gray dashed curve demonstrates the average ρ_{ab}. Please note that the coloring scale is different on each graph, to clarify the discrepancy between different regions of each graph.
