# Supplementary material for: Impact of temporal correlations on high risk outbreaks of independent and cooperative SIR dynamics
Source: PLoS One. 2021 Jul 20;16(7):e0253563. doi: 10.1371/journal.pone.0253563 (PMC8291698; doi:10.1371/journal.pone.0253563)
Supplement: S2 Appendix — (PDF) [file pone.0253563.s002.pdf]

S2 Appendix. k-means algorithm and illustration

The k-means procedure is described in Algorithm 1.

Algorithm 1 k-means algorithm

```
1: Initialize Centroids.
2: repeat
3:     Assign data points to the cluster of their closest centroid.    ▷ Label Assignment
4:     Set centroids to the mean of their associated clusters.    ▷ Centroid Calculation
5: until Centroids converge.
```

The initial positions of the centroids (Line 1) can be chosen randomly, or via algorithms such as k-means++ [1]. Convergence criteria (Line 5) is met when centroid positions, and therefore clusters cease to alter.

In Fig. 1, we have illustrated the progress of the k-means algorithm, performed on the DCWB shuffled hospital network with $q = 1$ and $r = 0.001$ simulation results.

[1] Wikipedia contributors. K-means++ — Wikipedia, The Free Encyclopedia; 2021. https://en.wikipedia.org/w/index.php?title=K-means%2B%2B&oldid=1013985307.

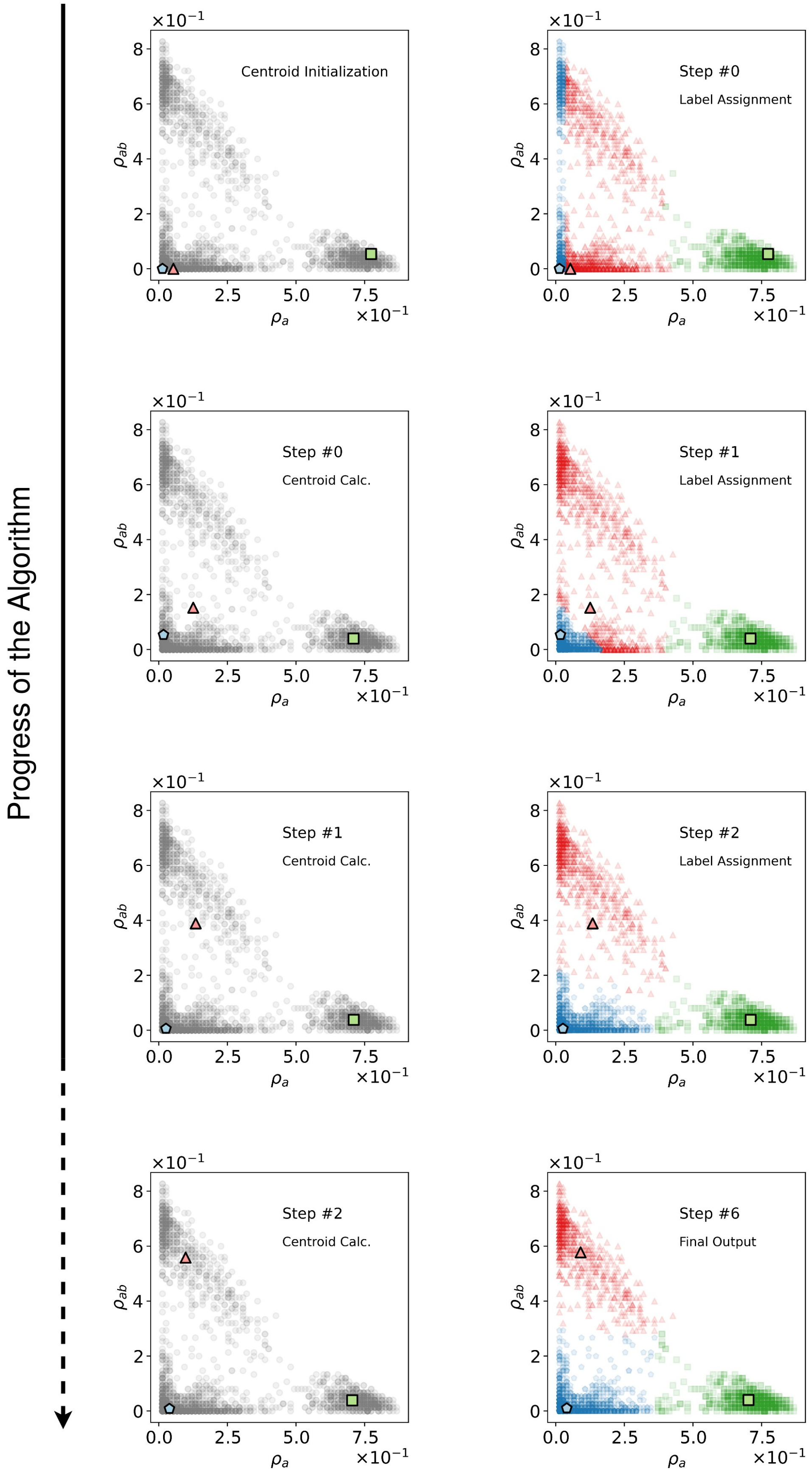

FIG. 1: Illustration of the progress of the k-means algorithm, performed on the DCWB shuffled hospital network with $q = 1$ and $r = 0.001$ simulation results. Larger markers represent centroids. Each small marker denotes a single simulation realization (data point). Their colors and shapes indicate association with their clusters/centroids. Gray circles represent unassigned data points. The procedure is described in Algorithm 1. Centroid Initialization panel illustrates Line 1 where centroids are defined. Label Assignment panels demonstrate Line 3 where each data point is assigned to the cluster of their closest centroid. Centroid Calc. panels illustrate Line 4 where centroids are positioned at the center of their associated clusters.
